# Diversity of sponge mitochondrial introns revealed by *cox 1 *sequences of Tetillidae

**DOI:** 10.1186/1471-2148-10-288

**Published:** 2010-09-20

**Authors:** Amir Szitenberg, Chagai Rot, Micha Ilan, Dorothée Huchon

**Affiliations:** 1 Department of Zoology, Tel-Aviv University, Tel Aviv 69978, Israel; 2 Department of Molecular Genetics, Weizmann Institute of Science, Rehovot 76100, Israel; 3 National Evolutionary Synthesis Center, 2024 W. Main St., Suite A200, Durham, NC 27705, USA

## Abstract

**Background:**

Animal mitochondrial introns are rare. In sponges and cnidarians they have been found in the *cox 1 *gene of some spirophorid and homosclerophorid sponges, as well as in the *cox 1 *and *nad 5 *genes of some Hexacorallia. Their sporadic distribution has raised a debate as to whether these mobile elements have been vertically or horizontally transmitted among their hosts. The first sponge found to possess a mitochondrial intron was a spirophorid sponge from the Tetillidae family. To better understand the mode of transmission of mitochondrial introns in sponges, we studied *cox 1 *intron distribution among representatives of this family.

**Results:**

Seventeen tetillid *cox 1 *sequences were examined. Among these sequences only six were found to possess group I introns. Remarkably, three different forms of introns were found, named introns 714, 723 and 870 based on their different positions in the *cox 1 *alignment. These introns had distinct secondary structures and encoded LAGLIDADG ORFs belonging to three different lineages. Interestingly, sponges harboring the same intron form did not always form monophyletic groups, suggesting that their introns might have been transferred horizontally. To evaluate whether the introns were vertically or horizontally transmitted in sponges and cnidarians we used a host parasite approach. We tested for co-speciation between introns 723 (the introns with the highest number of sponge representatives) and their nesting *cox 1 *sequences. Reciprocal AU tests indicated that the intron and *cox 1 *tree are significantly different, while a likelihood ratio test was not significant. A global test of co-phylogeny had significant results; however, when cnidarian sequences were analyzed separately the results were not significant.

**Conclusions:**

The co-speciation analyses thus suggest that a vertical transmission of introns in the ancestor of sponges and cnidarians, followed by numerous independent losses, cannot solely explain the current distribution of metazoan group I introns. An alternative scenario that includes horizontal gene transfer events appears to be more suitable to explain the incongruence between the intron 723 and the *cox 1 *topologies. In addition, our results suggest that three different intron forms independently colonized the *cox 1 *gene of tetillids. Among sponges, the Tetillidae family seems to be experiencing an unusual number of intron insertions.

## Background

Mitochondrial introns are self-splicing, selfish and mobile genetic elements [1-3]. The mobility of these introns is often facilitated by homing endonucleases (HEs) that are encoded within the introns [4,5]. Mitochondrial introns are rare in Metazoa. Both group I and group II introns have been described. Group II introns are the least frequent. They have only been found in Placozoa [6] and in an annelid worm [7]. Group I introns have been found in several unrelated Cnidaria (e.g. [8-11]), Porifera (e.g. [12,13]), and Placozoa (e.g., [6]). As a case in point, *Tetilla *sp. SP25456 (Spirophorida, [12]) and *Plakinastrella onkodes *(Homosclerophorida, previously identified as *Plakortis angulospiculatus *[13], D. Lavrov personal communication) are the only two sponges found to possess mitochondrial introns, although 22 complete mitochondrial genomes, representing a wide demosponge diversity, have already been sequenced [13-17]. A recent study of the Lebanon sponge fauna suggests that *Tetilla *sp. SP25456 should be synonymized with *Cinachyrella levantinensis *[18]. To confirm this view we sequenced a 1650 bp fragment of the 18S rRNA for both a *Tetilla *sp. sample from Israel and a *C. levantinensis *sample from Lebanon (Additional file 1). The same sequence was obtained for both samples. Consequently, we use here the name *C. levantinensis*, rather than *Tetilla *sp. SP25456.

The *C. levantinensis cox 1 *intron was found to be 1,138 bp long [12]. Unfortunately, the *cox 1 *sequence of *C. levantinensis *is not complete. Using the complete *cox 1 *sequence of *Amphimedon queenslandica *[19] as reference, the *C. levantinensis *intron was found to be inserted after position 723. This intron encodes a putative LAGLIDADG protein. In *P. onkodes *two group I introns were found in the *cox 1 *gene but their secondary structures were not provided [13]. These introns are 388 bp and 1,118 bp in size, and are separated by 9 nucleotides (3 codons). The second intron of *P. onkodes *is inserted at the same position as the intron reported for *C. levantinensis *[13]. This intron and its counterpart in *C. levantinensis *share 81.2% nucleotide sequence identity, have a similar secondary structure, and contain LAGLIDADG ORFs. By contrast, they share only ~43% sequence identity with the first intron of *P. onkodes*, which does not contain any ORF [13].

We have previously shown that the *C. levantinensis *intron was more closely related to fungi introns than to any animal intron known at that time [12]. Hence we suggested that the presence of this intron in a sponge may be the result of a horizontal gene transfer event between fungi and sponges [12]. Based on a later discovery of a highly similar intron inserted at the same position in 20 scleractinian corals of the suborder Faviina [9] and in the sponge *P. onkodes*, [13] Fukami et al. [9] and Wang & Lavrov [13] concluded, in contrast to our hypothesis, that this intron had most likely been transmitted vertically in cnidarians and sponges, but independently lost in most lineages. However, no statistical analyses were conducted in those studies, and it is thus difficult to determine which hypothesis is better supported by the data. Interestingly, using reciprocal Shimodaira-Hasegawa (SH) tests, a Bayes factors test for incongruence, and a non-parametric version of Huelsenbeck and Bull's likelihood ratio test, Goddard et al. [10] showed that another intron, not related to the *C. levantinensis *intron and inserted at a different position, was horizontally transferred among actinarian cnidarians.

In order to better understand the origin and evolution of sponge mitochondrial introns we determined the *cox 1 *gene of 15 tetillid sponges and present statistical evidence that the *C. levantinensis *intron was both horizontally and vertically transferred in animals.

## Results

### Presence of mitochondrial introns and insertion sites

**Figure 1:**
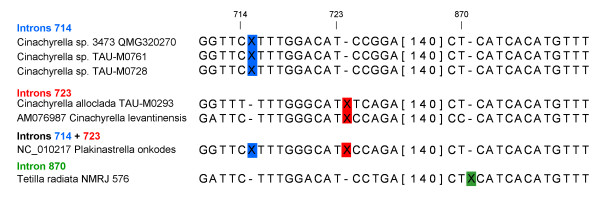
**Intron insertion sites**. Insertion sites of introns 714, 723 and 870 found in sponges are denoted by X. The *cox 1 *sequence of the sponge *A. queenslandica *was used as reference for numbering positions in the alignment.

Fifteen new and two existing tetillid *cox 1 *sequences from four genera were considered in our analysis: *Cinachyrella *(9 specimens/6 species), *Craniella *(five specimens/4 species) *Paratetilla *(one specimen) and *Tetilla *(two specimens/two species). To avoid bias we only considered sequences that included all the insertion site positions of both the previously discovered and newly discovered tetillid introns. However, since we did not amplify complete *cox 1 *sequences, introns present at the beginning (the first 108 - 156 bp are missing, depending on the sequence) or at the end of the gene (the last 615 - 1074 bp) would not be detected by our study. The complete *cox 1 *sequence of the sponge *A. queenslandica *was used as reference for numbering positions in the alignment. The use of reference sequence allowed a comparison of insertion sites between *cox 1 *CDSs of different lengths, as well as between partial and complete sequences. Among the 15 new sequences only five were found to possess a longer *cox 1 *sequence, most probably indicating the presence of introns. Only the *Cinachyrella alloclada *sample was found to have an 1140 bp long insertion after position 723. This insertion is similar in length and insertion site to those previously found for the intron of *C. levantinensis *and the second intron of *P. onkodes *(introns 723, Figure 1). All other insertions are shorter and inserted at different positions of the alignment. Two *Cinachyrella *sp. samples from Zanzibar as well as the *Cinachyrella *sp. 3743 sample from Australia (which probably belongs to the same species), have a 985 bp long insertion that is inserted after position 714 (introns 714, Figure 1). This insertion site is similar to that of the first intron of *P. onkodes*. Finally, the *Tetilla radiata *sample has a 948 bp long insertion that is inserted after position 870 (intron 870, Figure 1). Interestingly, the introns present in the cnidarians *Palythoa *sp. and *Savalia savaglia *are also inserted at this position. A BLASTX analysis [20] of the inserted sequences allowed us to identify a single LAGLIDADG ORF in each insertion.

### Intron secondary structures and sequence similarity

**Figure 2:**
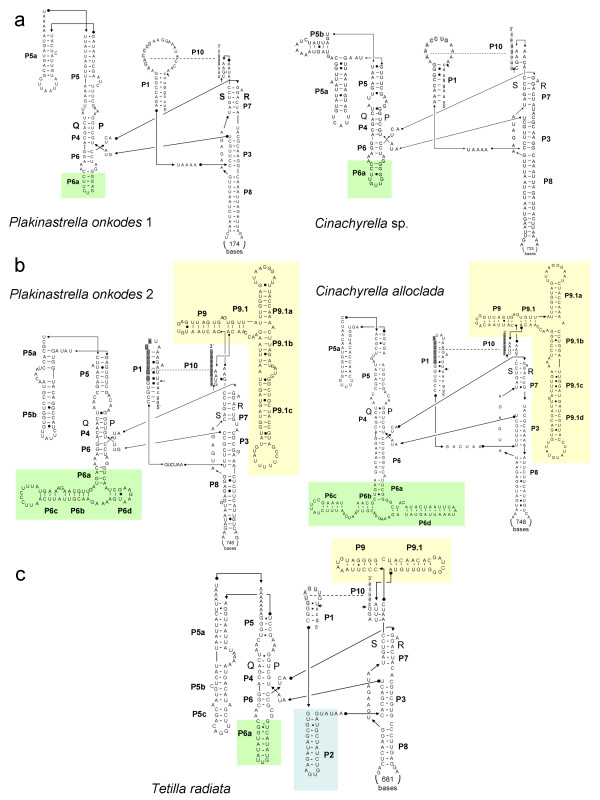
**Secondary structures of representative group I introns in sponges**. The secondary structure of introns 714 (a) is represented by *Cinachyrella *sp. TAU-M0728 and the first intron of *P. onkodes *NC_010217. The secondary structure of introns 723 (b) is represented by the sponge *C. alloclada *TAU-M0293 and the second intron of *P. onkodes *NC_010217. The secondary structure of intron 870 is represented by *T. radiata *NMRJ-576.

All inserted sequences were found to fold into the canonical group I intron secondary structure, confirming that the insertions are indeed introns. However, not all introns had the same secondary structure. Three different secondary structures, corresponding to introns 714, 723 and 870, were reconstructed from the five sequences (Figure 2). Introns 723 are represented in sponges by the introns of *C. levantinensis*, *C. alloclada *and the second intron of *P. onkodes*. They are characterized by an absence of P2 as well as large P6 (69-96 bp) and large P9 (113-116 bp) regions (Figure 2b, [12]). Introns 714 were identified in the three *Cinachyrella *sp. samples from Zanzibar and Australia. The first intron of *P. onkodes *was also identified as an intron 714. These introns are characterized by an absence of P2 and P9 as well as the presence of a large loop (> 10 bp) in their P1 structure (Figure 2a). It should be noted that the *Cinachyrella *sp. and *P. onkodes *intron 714 structures differ in their P5 region, which is longer in *Cinachyrella *sp. Finally, an intron 870 was only found in the *T. radiata *sequence (Figure 2c). This last intron is characterized by the presence of a P2 and a longer P5 than other sponge introns. In all introns most of the LAGLIDAGD ORF is located in the loop associated with the paired region P8. Each LAGLIDADG ORF was found to contain at least one UGA codon. This codon corresponds to a stop codon in the standard genetic code but to tryptophan in the "mold, protozoan, and coelenterate" mitochondrial code (i.e., the demosponge mitochondrial code), refuting the possibility of a nuclear origin for the introns. It is worth noting that most mitochondrial genetic codes could code for a LAGLIDADG sequence. It is thus not possible to determine the origin of the intron based on a genetic code. High sequence similarity exists among the introns inserted at the same position. The percentage of identity ranges from 85 to 99% between two sponge introns inserted at the same position, and from 43 to 53% between introns with different insertion sites.

### The LAGLIDADG phylogeny

**Figure 3:**
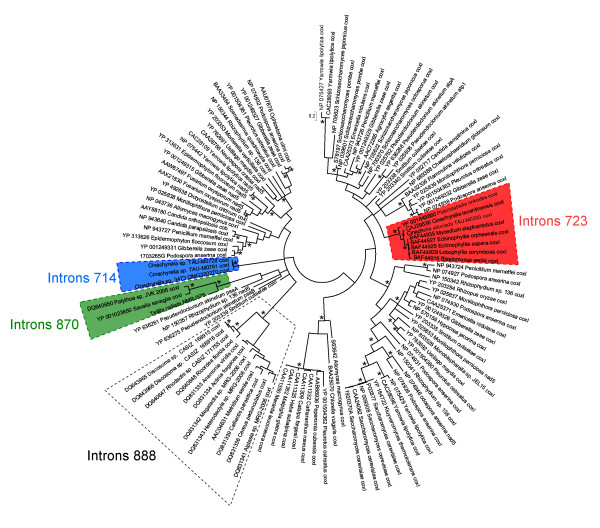
**Phylogenetic tree reconstructed based on LAGLIDADG protein sequences**. Bayesian phylogenetic tree reconstructed from LAGLIDADG protein sequences nested within group I introns of fungi, chlorophytes, streptophytes, cnidarians and sponges. Metazoan LAGLIDADG clades from introns 714, 723, 870 and 888 are delineated with a dashed line. Asterisks denote PP > 0.95 and BP > 90. NCBI Protein accession numbers are indicated.

To identify the evolutionary relationships of the LAGLIDADG sequences encoded within the three intron forms, as well as their putative origin, a phylogenetic tree was reconstructed based on the LAGLIDADG protein sequences encoded within sponge introns together with similar sequences identified by a BLASTP search [21] (see Methods section). The endonuclease ORF encoded within the intron and the intron section that takes part in creating the intron's secondary structure can have different histories [1]. In this analysis, the section of the intron involved in creating its secondary structure was excluded. Consequently, the phylogenetic result allowed us to establish whether the LAGLIDADG sequences encoded within introns of similar structure and insertion sites are closely related. The resulting tree was unrooted (Figure 3). The first intron of *P. onkodes *was not included in this analysis since it lacked an ORF. The LAGLIDADG sequences of sponges belonged to three unrelated clades corresponding to the different insertion sites and secondary structures, which suggest that the LAGLIDADG ORFs were transmitted with their introns. Interestingly, not only the clade comprising the ORFs of introns 723 (cf. [9,12,13]) but also the clade comprising the ORFs of introns 870 included cnidarian representatives (i.e., the corals *Palythoa *sp. and *S. savaglia *[11], which had an intron inserted at the same position as *T. radiata*). The clade comprising the ORFs of introns 714, in contrast, was not found to be related to any cnidarian sequence. Another unrelated animal clade included only cnidarian sequences. The LAGLIDADG sequences of this clade originated from introns inserted at position 888. Goddard et al. [10] studied the co-phylogeny of the LAGLIDADGs encoded within introns 888 and their host.

None of the animal LAGLIDADG sequences were found to be closely related (i.e. similarity below 65%) to any fungi or plant sequence.

### The *cox 1 *phylogeny

**Figure 4:**
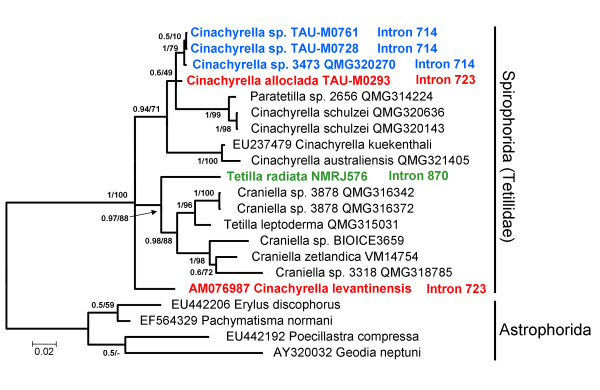
**Phylogenetic tree reconstructed based on *cox 1 *coding sequence**. Bayesian tree reconstructed from the *cox 1 *sequences of tetillids and an outgroup (astrophorid sponges). Introns are indicated next to the sequences possessing them. Posterior probabilities/ML bootstrap supports are indicated at the base of each branch. Accession numbers are indicated prior to the name of the taxon.

To identify whether closely related introns were hosted by closely related sponges, a phylogenetic tree was reconstructed based on *cox 1 *sequences (Figure 4) using the maximum likelihood (ML) criterion under the TrN + Γ + I model and Bayesian analyses under the CAT + Γ4 site-heterogeneous model [22]. The monophyly of Tetillidae was recovered with maximum support (Bayesian posterior probability: PP = 1.0; and ML bootstrap percentage: BP = 100). In contrast, most of the represented tetillid genera were found to be paraphyletic. More specifically, the representative of *Paratetilla *was found to be closely related to *Cinachyrella *species. Similarly, the sequences of *Tetilla leptoderma *and *Craniella *sp. 3878 were found to be more closely related to each other than to other representatives of their respective genera. These observations indicate the need for a closer examination of the Tetillidae phylogeny, using additional markers and a larger taxon sampling. Surprisingly, *cox 1 *sequences possessing introns did not always cluster together. While the three *Cinachyrella *spp. specimens possessing introns 714 formed a monophyletic clade, *C. alloclada *and *C. levantinensis*, which possessed introns 723, belong to distant lineages.

### Co-speciation between *cox 1 *sequences and their nesting LAGLIDADG sequences

In order to examine whether the sponge introns were transmitted vertically, we checked for co-evolution between *cox 1 *coding sequences (CDSs) and intron sequences (the sequences included both the LAGLIDADG and the non-coding regions involved in the intron secondary structure) nesting within the *cox 1 *genes. Such methods are usually used to explore co-speciation between two organisms (e.g., host - parasite relationship). In the case of *cox 1 *sequences and their introns it is indeed possible to consider the introns as parasites of the *cox 1 *genes. Since in Tetillidae we have three unrelated "parasitic" introns (714, 723, 870), the history of each intron should be considered separately. Because only a few species were found to possess an intron 714 or an intron 870, our co-speciation analyses were only based on introns 723. The *cox 1 *and intron 723 sequences of 20 corals and three sponges possessing such introns were considered in these analyses.

**Figure 5:**
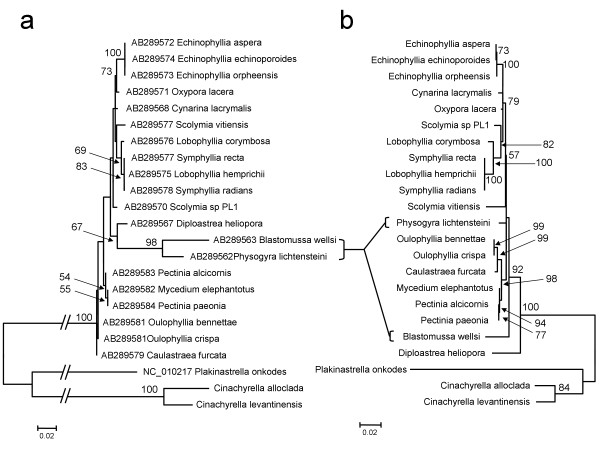
**Phylogenetic trees of taxa possessing intron 723**. ML trees reconstructed from the *cox 1 *(a) and intron (b) sequences. Accession numbers are indicated prior to the name of the taxon. BP > 50 computed with the program PhyML 3.0 [59] are shown at the base of nodes.

Following Goddard et al. [10], we first conducted tests of competing evolutionary hypotheses. More specifically, using reciprocal approximate unbiased (AU) tests we evaluated, for each data set separately whether the likelihood scores obtained under the intron ML topology (Figure 5a) were significantly different from the likelihood scores obtained under the *cox 1 *ML topology gene (Figure 5b). Both the *cox 1 *and the intron data sets reject the H_0 _hypothesis that the two genes could share the same topology (*p *< 0.001).

Because reciprocal AU tests are more adapted when node support is high [10] we also applied a non-parametric version of Huelsenbeck and Bull's likelihood ratio test for detecting conflicting signals [10,23]. Like the AU tests, the LRT test assumes an identical phylogeny for the *cox 1 *and LAGLIDADG genes. However, in this test both data sets are not considered separately. The test computes the difference between the likelihood scores obtained when both data sets have different topologies ('two trees model') and the likelihood scores obtained when both data sets have the same topology ('one tree model'). This difference is then compared to a null distribution generated by non-parametric bootstrapping (see Methods). Unlike the AU tests, the likelihood ratio test does not reject the H_0 _hypothesis that the one-tree hypothesis (both genes have the same topology) is favored over the two-tree model (each gene has a different topology), although marginal significance is observed (*p *= 0.072; out of the 500 replications performed 36 were found to have a smaller log-likelihood ratio statistic than the original data set, Additional file 2).

The fact that the reciprocal AU tests reject the hypothesis of co-phylogeny and that the LRT test is marginally significant indicates the presence of at least one incongruent node between the LAGLIDADG and *cox 1 *trees. These results support the hypothesis of horizontal gene transfer of introns. However they do not exclude the possibility that in some clades a vertical transmission is the most likely hypothesis. We therefore also conducted a global test of co-evolution, as well as a test on each host parasite link, using the program ParaFit [24]. In this approach, matrix permutations are used to compare *cox 1 *and intron patristic distances. The analysis is thus unconstrained by the phylogenetic tree. Unlike the AU and likelihood ratio tests, the null hypothesis in ParaFit is that the host and parasite phylogenies are randomly associated, and thus that both data sets assume a different topology. Hence, a significant *p*-value indicates the existence of at least one congruent host-parasite link between the LAGLIDADG and *cox 1 *trees. In such a case, the tests on each host parasite link allow us to identify the co-speciating host-parasite pairs. This test revealed that the global co-speciation parameter, was significant (*p *< 0.001), albeit low (ParaFitGlobal = 0.0047). Only 3 out of 23 pairwise co-speciation links examined were significant, and again the link values were low (0.0019 < ParaFitLink1 < 0.002, 0.001 <*p *< 0.006). These three significant links represented the relationships between each of the sponges sampled and their intron sequences. When these three taxa were removed from the analysis, the global test indicated no co-speciation (ParaFitGlobal = 0.00001, *p *> 0.05). The ParaFit result thus suggests that intron 723 could have been vertically transferred in tetillid and homoscleromorph sponges. However, this result is most probably an artifact due to the fact that only three sponges, belonging to different distant classes, were considered in this analysis. In support of such an idea, it is worth noting that the two tetillid LAGLIDADG proteins are closer to the cnidarian sequences than to the homoscleromorph sequence (PP = 0.98, BP = 80, Figure 3). The latter result contradicts the current view that sponges are either monophyletic [25] or that homoscleromorphs are closer to cnidarians [26]. It seems thus more appropriate to explain the distribution of intron 723 in homoscleromorphs and tetillids by at least two independent transfer events. However, we cannot exclude the possibility that this intron has been vertically transmitted in tetillids.

## Discussion

Mitochondrial introns are rare among metazoan organisms. Surprisingly, more than a fifth of the Tetillidae species considered in our study (4 species out of 13) were found to possess mitochondrial introns. This indicates an unusually high number of introns within this family. More surprisingly, our findings pointed to the existence of three different introns within the tetillid family. Indeed, each intron form has a different insertion site and secondary structure, and the LAGLIDADG they encode belong to unrelated clades.

There are two characteristics that might explain the presence of introns in tetillid sponges. First, sponges, similar to cnidarians, have a slower mitochondrial evolutionary rate than bilaterians [14,27]. This slow evolutionary rate has been suggested to facilitate the proliferation of group I introns since their splicing depends on the conservation of a rather large sequence of nucleotides (n > 20) [28,29]. Second, the transmission of genetic material is not restricted to the germline in tetillids due to their regeneration capacity [30] and budding ability [31,32]. Therefore, an intron acquired in any somatic cell has the potential to be transmitted to future generations. Interestingly, although not all members of the phylum Porifera reproduce asexually, homoscleromorphs, the second sponge lineage known to possess mitochondrial introns, also have a budding capacity [33].

Our results suggest that the family Tetillidae is a hot spot for the presence of group I introns in animals. However, we cannot exclude the possibility that introns might be overlooked in other sponge lineages. Indeed, two of the discovered introns are located within the reverse primer that has been recommended for the amplification of the barcoding region of the *cox 1 *gene in sponges [34]. As a case in point, Cárdenas et al. [35] failed to amplify the *cox 1 *sequence of *C. alloclada *using those primers, whereas we successfully amplified this species using different primers. This suggests that "standard" barcoding primers might not be adapted for the sponge and cnidarian species that share introns 714 and 870. It is therefore likely that other sponge lineages might contain mitochondrial introns, in particular those with a budding ability, for example, members the genus *Tethya *[36,37].

Two main scenarios can explain the distribution of these introns: one includes only vertical transmission while the second incorporates events of horizontal transfers. A scenario that includes only vertical transmission, as suggested by Wang & Lavrov [13], would imply that the ancestor of sponges and cnidarians possessed no less than four different introns in its *cox 1 *sequence (Figure 3). The fact in favor of this hypothesis is that both introns 723 and 870 are shared by sponges and cnidarians. However, no species has been found to possess all four introns. Except for *P. onkodes *which possesses introns 714 and 723, all other individuals with introns in their *cox 1 *sequence possess only a single intron. Since tetillids belong to the largest group of demosponges (the G4 clade, [38,39]), the hypothesis of a vertical transmission of the introns implies a tremendous number of independent losses in most demosponge lineages, but not in Tetillidae, whose ancestor retained three introns that were later independently lost in most tetillid species. Clearly, this scenario is improbable.

Various facts support instead the occurrence of horizontal transfer events within the mitochondrial genome of sponges. First, group I introns are known to be invasive elements that independently colonized the mitochondrial genome of numerous plants and fungi [3]. Second, the reciprocal AU tests (or SH tests [10]) on intron 723 (see Results) and intron 888 [10] support the idea that the *cox 1 *and intron topologies are significantly different rejecting the hypothesis of co-phylogeny. Based on the ParaFit results none of the host-parasite link is significant within Faviina corals, supporting the absence of co-phylogeny. The ParaFit results are not affected by either the node supports or the topology of the studied genes. It is possible that our ParaFit results are affected by differences in molecular-evolutionary rates between the two genes. Indeed, the *cox 1 *sequences of the coral *Blastomussa wellsi *and *Physogyra lichtensteini *appear to have evolved at a faster rate than other coral species (Figure 5a), although this result might be the consequence of a misplacement of the root [9]. In contrast, the evolutionary rate of these four genera is not at odds with those of the other intron sequences (Figure 5b). Such rate differences between *cox 1 *and LAGLIDADG could affect the ParaFit conclusions, if they are not the result of a different history for each gene [40]. However, there are several points in favor of true phylogenetic differences between the genes. First, reciprocal AU-tests, which do not take branch-length into account, reject the hypothesis of co-phylogeny (the AU-tests are also significant in the absence of sponge sequences, data not shown). Second, Fukami et al. [9] noticed an incongruence between the *cox 1 *and LAGLIDADG topologies and acknowledged the possibility of horizontal transfers in scleractinian. It is therefore likely that the differences between the evolutionary rates of *cox 1 *and LAGLIDADG indicate a different lateral gene-transfer in *Blastomussa wellsi *and *Physogyra lichtensteini*.

Finally, although the LAGLIDADG tree suggests that the sponge and cnidarian introns have an ancient fungal origin, the specific donor of the Porifera introns is still unknown. No sequence except for cnidarian was found to be closely related to any of the sponge introns. A transfer from a cnidarian to a sponge or from a sponge to a cnidarian, as first suggested by Fukami et al. [9], appears unlikely in the case of Tetillidae since these sponges are mainly found in sediment habitats [41] and not in close proximity to corals. It is more likely that the same donor (e.g., a fungus) was at the origin of the sponge and cnidarian intron.

## Conclusions

Our results support the suggestion that mitochondrial introns are both horizontally and vertically transferred in sponges and cnidarians. Given the absence of a complete phylogenetic resolution of the *cox 1 *and LAGLIDADG tree, the specific cases in which these introns were horizontally *versus *vertically transmitted still remain to be determined. Among sponges, the family Tetillidae appears as a hot spot of intron insertions, with three different intron forms present in different individuals. Interestingly, two intron forms found in sponges are closely related to cnidarian introns, suggesting that sponge and cnidarian introns might originate from a similar donor. However, the mechanisms and the donor at the origin of this transfer of genetic material remain to be discovered.

## Methods

### Samples and molecular manipulations

The *cox 1 *gene of 15 tetillid sponges was amplified. The origin of the samples is indicated in Additional file 3. The DNA of each sample was isolated using a modified PVP protocol [42]. The *cox 1 *sequences were amplified using two step nested PCRs. The conditions of PCR amplifications were: 94°C for 2 min; 35 cycles at 94°C for 50 sec, 50°C for 50 sec, 72°C for 4 min; and a final elongation at 72°C for 10 min. Different primer pairs were used depending on the species considered. The list of primer used and their sequences are indicated in Additional file 4. Amplified fragments were directly sequenced on an ABI PRISM 3100 (Applied Biosystems) genetic analyzer. The sequences were submitted to GenBank under accession numbers HM032738-HM032752.

### Characterization of the intron sequences

Intron insertion-sites were determined manually, based on nucleotide and amino-acid alignments of *cox 1 *sequences. The core structure of the *cox 1 *introns was determined with Citron [43]. Peripheral hairpin structures were predicted with Mfold [44,45] using default setting. Finally, the graphic visualizations of the secondary structures were generated with RnaViz [46]. ORFs were sought for within each intron sequence. For each ORF identified, a BLASTX search [20] was conducted to determine the protein family it belonged to, if any.

### Phylogenetic reconstruction based on LAGLIDADG protein sequences

Following Rot et al. [12], a LAGLIDADG dataset was constructed using BLASTP searches. Each of the LAGLIDADG sequences found in sponge introns was used as query. This dataset included mostly fungi, as well as plants, cnidarians and sponges. GenBank accession numbers are indicated in Figure 3. The protein sequence alignment was conducted with the L-INS-i algorithm under the JTT-200 substitution matrix, as implemented in Mafft version 6 [47]. Due to the high variability of the LAGLIDADG sequences, the use of Gblocks [48] or Soap [49] to remove poorly aligned region of the alignment, resulted in matrices less than 100 amino-acids (aa) long. The LAGLIDADG data set was therefore corrected manually. Sections of the alignment with more than one third of missing data were removed. Additionally, the 5' region of the LAGLIDADG ORF which takes part in the folding of the intron (i.e., the first 63 aa positions of the LAGLIDADG alignment that correspond to the largest such region) were removed as well. The final LAGLIDADG data set included 217 characters; 3 of which were constant (Additional file 5). All of the 214 variable characters were phylogenetically informative. The reconstruction of the tree was conducted with RAxML 7.0.4 [50] with 100 bootstrap repeats, under the CAT + Γ + I, and with Phylobayes 3.2 [51] under the CAT model. The Phylobayes analysis included two chains with 18,100 cycles (3,400,000 generations) while 4,500 cycles were discarded as burnin. The *maxdiff *value of this run was 0.069.

### Phylogenetic reconstruction based on *cox 1 *CDS

The *cox 1 *data set included the 15 DNA sequences obtained as well as two tetillid sequences available in GenBank. Since Astrophorida has been shown to be the sister clade of Spirophorida (e.g. [38,39]), four astrophorid sequences were used as outgroup. Accession numbers are indicated in Figure 4. A codon alignment of the *cox 1 *sequences was obtained using the online version of Pal2Nal [52]. The underlying protein reference data set was aligned using MAFFT version 6 [47] with the L-INS-i algorithm. The program Gblocks [48] was then used to exclude regions that were poorly aligned. The nucleotides downstream to the intron insertion site (i.e., 18 nucleotides after each insertion) were also removed since co-conversion of *cox 1 *exonic sequences can occur after intron insertion [53]. The *cox 1 *data set used in the phylogenetic analysis included 927 characters; 687 of them were constant and 303 had missing data. Among the 240 variable sites 190 were phylogenetically informative (Additional file 6). Phylogenetic reconstruction was conducted using both the maximum likelihood (ML) and the Bayesian approaches. The ML tree was reconstructed with PAUP* 4 [54] under the TrN + Γ + I model of sequence evolution and using the tree bisection reconnection (TBR) branch-swapping algorithm and 100 random sequence addition starting trees. The TrN + Γ + I model was found to be the best fitting ML model using Modeltest 3.7 [55]. Branch supports were estimated based on 100 bootstrap repetitions. A Bayesian tree was reconstructed with Phylobayes 3.2c [51] under the GTR CAT model of sequence evolution. The analysis included two chains with a total run length of 13,000 cycles (490,000 generations) while 3200 cycles were discarded as burnin. The *maxdiff *value of this run was 0.038.

### Co-speciation between "host" (*cox 1 *coding sequences) and "parasite" (introns 723)

Three different introns were found in sponges (see Results section). However, co-speciation tests, between intron sequences and their *cox 1 *host sequence, could only be performed for intron 723. Other introns did not include enough representatives, except intron 888, which was already studied by Goddard et al. [10].

A total of 20 species of corals and three species of sponges possessing intron 723 were considered. The nucleotide data sets of each gene were aligned with MAFFT version 6 [47] with the L-INS-i algorithm. After manual removal of ambiguously aligned positions the *cox 1 *and intron data sets were respectively 630 and 1078 bp long (Additional files 7, 8. The program PAUP* 4 [54] was used to reconstruct the evolutionary relationships based on *cox 1 *and intron sequences, and to obtain the corresponding matrices of patristic distances. The two ML phylogenetic trees were obtained using the TBR branch-swapping algorithm under the best model of sequence evolution identified by Modeltest 3.7 [55].

Three co-speciation tests were conducted. Following Goddard et al. [10] the first approach was to compare the ML topology of both genes under each data set. Reciprocal approximate unbiased tests as implemented in Consel 0.1i [56] were applied. The site-wise log-likelihoods were obtained using PAUP* 4 [54]. The second approach was to perform a non parametric version of Huelsenbeck and Bull's likelihood ratio test [23]. This test compares the log-likelihood *L_0 _*obtained under the hypothesis that the *cox 1 *gene and the intron share a single tree (each marker being allowed to evolve under different model and branch-length parameters), with the log-likelihood *L_1 _*obtained under the hypothesis that each data set evolved independently, resulting in different trees and sets of parameters. Computations were performed with PAUP* under the GTR + Γ + I model for both gene. The log-likelihood ratio statistic (*d*) is defined as:

$$d=2(\ln L_{0}-\ln L_{1})$$

The null distribution of *d *was obtained using non-parametric bootstrap. Five hundred bootstrap files were generated separately for the *cox 1 *and the intron data sets using Mesquite 2.72 [57]. Each *cox 1 *bootstrap file was concatenated with one LAGLIDADG bootstrap file. The *cox 1 *and intron sequences were then randomized within each concatenated file using a Perl script designed for this purpose. Three ML trees were constructed for each data set. The first was based on the first 630 positions of the concatenated and randomized matrix, the second on the last 1078 positions and the third on the total matrix length (1708 bp). The tree reconstructions were performed with PAUP* using rounds of heuristic searches starting with a neighbor-joining (NJ) tree and using tree bisection-reconnection (TBR) branch-swapping. The initial model parameter values were those estimated by Modeltest. After a first round of heuristic search the parameters were estimated on the resulting tree and then used for the subsequent round of heuristic search. The process was repeated until all parameters converged. Ln *L_0 _*was computed by assuming that the first 630 bp and the last 1078 bp shared the ML tree obtained using the whole data set (albeit each partition was allowed to evolve under different model and branch-length parameters). Ln *L_1 _*was computed by assuming that the first 630 bp and the last 1078 bp had different trees and different models of evolution. The third approach applied the program ParaFit [24] to perform a global test of co-speciation (ParaFitGlobal) as well as local tests for each *cox 1 *- intron link. Following the recommendation of the ParaFit manual, the only parameter considered was ParaFitLink1. This parameter is indeed more adapted when the global test is significant but the local tests show a mixed trend. The principle coordinates (PCOs) of the patristic matrices were calculated with DistPCoA [58] using the Lingoes correction.

## Authors' contributions

DH conceived the study and supervised the sequencing and analyses. MI collected and identified the biological specimens. AS and CR carried out the sequencing. AS conducted the phylogenetic analysis. AS and DH drafted the manuscript. All other authors assisted in writing the manuscript. All authors read and approved the final manuscript.

## Supplementary Material

Additional file 1 **18S rRNA sequences of *Tetilla *sp. from Israel and *Cinachyrella levantinensis *from Lebanon**. DNA sequences alignment in nexus format. Click here for file

Additional file 2 **The null distribution of the log-likelihood ratio statistic (*d*)**. The null distribution of the log-likelihood ratio statistic (*d*) generated by the LRT analysis of co-evolution. Click here for file

Additional file 3 **Origin of the tetillid samples used in this study**. A table describing the voucher number, GenBank accession number, geographical origin and contributor of each sample sequenced in this study. Click here for file

Additional file 4 **PCR primers used in this study**. The sequences of PCR primers and their utilization in this study, specified for each specimen, are described in this file. Click here for file

Additional file 5 **LAGLIDADG protein alignment**. Protein sequence alignment (in Nexus format) used to reconstruct the phylogenetic tree present in Figure 3. Click here for file

Additional file 6 ***Cox 1 *DNA alignment**. DNA sequence alignment (in Nexus format) used to reconstruct the phylogenetic tree present in Figure 4. Click here for file

Additional file 7 **DNA alignment of the *cox 1 *sequences from taxa possessing introns 723**. C*ox 1 *sequence alignment in Nexus format used in the co-evolution analyses. Click here for file

Additional file 8 **DNA alignment of the sponge and cnidarian introns 723**. Intron sequence alignment in Nexus format used in co-evolution analyses. Click here for file
