# Supplementary figures and images for: Diversity of sponge mitochondrial introns revealed by cox 1 sequences of Tetillidae
Source: BMC Evol Biol. 2010 Sep 20;10:288. doi: 10.1186/1471-2148-10-288 (PMC2955029; doi:10.1186/1471-2148-10-288)

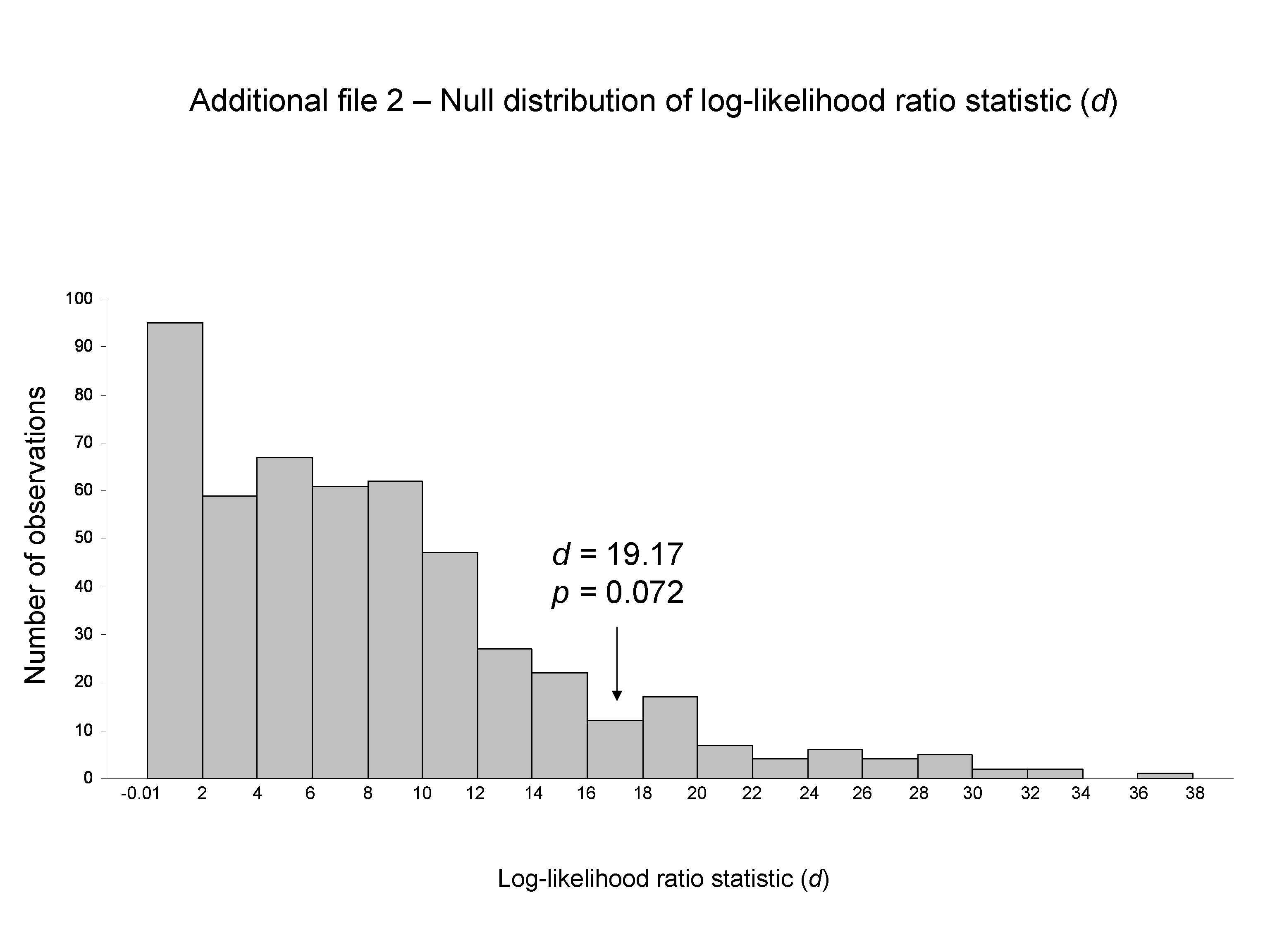

Supplement: Additional file 2 — The null distribution of the log-likelihood ratio statistic (d). The null distribution of the log-likelihood ratio statistic (d) generated by the LRT analysis of co-evolution. [file 1471-2148-10-288-S2.TIFF]
