# Supplementary material for: Diversity of sponge mitochondrial introns revealed by cox 1 sequences of Tetillidae
Source: BMC Evol Biol. 2010 Sep 20;10:288. doi: 10.1186/1471-2148-10-288 (PMC2955029; doi:10.1186/1471-2148-10-288)
Supplement: Additional file 3 — Origin of the tetillid samples used in this study. A table describing the voucher number, GenBank accession number, geographical origin and contributor of each sample sequenced in this study. [file 1471-2148-10-288-S3.DOC]

Additional File 3:

Tetillid samples used in this study

| **Specimen** | **Species** | **Intron** | **Origin** | **Donor/Collector** |
| --- | --- | --- | --- | --- |
| QMG314224 | *Paratetilla* sp 2656 | no | Australia | J. Hooper (Queensland Museum ) |
| QMG315031 | *Tetilla leptoderma* | no | Antarctica |
| QMG316342 | *Craniella* sp. 3878 | no | Australia |
| QMG316372 | *Craniella* sp. 3878 | no | Australia |
| QMG318785 | *Craniella* sp. 3318 | no | Australia |
| QMG320270 | *Cinachyrella* sp. 3473 | 714 | Australia |
| QMG321405 | *Cinachyrella australiensis* | no | Australia |
| QMG320143 | *Cinachyrella schulzei* | no | Australia |
| QMG320636 | *Cinachyrella schulzei* | no | Australia |
| BIOICE 3659 | *Craniella* sp. | no | Iceland | P. Cárdenas and H. T. Rapp (University of Bergen) |
| VM14754 | *Craniella zetlandica* | no | Iceland |
| NMRJ–576 | *Tetilla radiata* | 870 | Brazil | E. Hajdu (Museu Nacional/UFRJ, Rio de Janeiro) |
| TAU-M0293 | *Cinachyrella alloclada* | 723 | Bahamas | M.I. |
| TAU-M0728 | *Cinachyrella* sp*.* | 714 | Zanzibar |
| TAU-M0761 | *Cinachyrella* sp*.* | 714 | Zanzibar |
