# Supplementary material for: Diversity of sponge mitochondrial introns revealed by cox 1 sequences of Tetillidae
Source: BMC Evol Biol. 2010 Sep 20;10:288. doi: 10.1186/1471-2148-10-288 (PMC2955029; doi:10.1186/1471-2148-10-288)
Supplement: Additional file 4 — PCR primers used in this study. The sequences of PCR primers and their utilization in this study, specified for each specimen, are described in this file. [file 1471-2148-10-288-S4.DOC]

Additional file 4:

PCR primers used in this study

| Name | Sequence / Source |
| --- | --- |
| LCO -1490 | Folmer et al., 1994 |
| cox1 R1 | Rot et al. 2006 |
| cox1 calcD1 | 5'-TWTNTTCWACHAAYCAYAAAGAYAT-3' |
| cox1 calcR1 | 5'-AARAARTGTTGRGGGAARAADGT-3' |
| cox1 tet R1 | 5'-TARCAATANCCNGTDATTTTNCCRAATCA-3' |
| cox1 tet R11 | 5'-CCAGTWATTTTNCCGAATCAA-3' |

| Sample ID | Accession | Species | External PCR primers | Internal PCR primers |
| --- | --- | --- | --- | --- |
| BIOICE 3659 | HM032750 | *Craniella* sp. | LCO - cox1 calc R1 | LCO - cox1 tet R1 |
| VM14754 | HM032751 | *Craniella zetlandica* | LCO - cox1 calc R1 | LCO - cox1 tet R1 |
| NMRJ-576 | HM032742 | *Tetilla radiata* | cox1 calc D1 - cox1 calc R1 | LCO - cox1 R1 |
| QMG314224 | HM032744 | *Paratetilla* sp. 2656 | LCO - cox1 calc R1 | LCO - cox1 tet R11 |
| QMG315031 | HM032749 | *Tetilla leptoderma* | LCO - cox1 calc R1 | LCO - cox1 tet R11 |
| QMG316342 | HM032747 | *Craniella* sp. 3878 | LCO - cox1 calc R2 |  |
| QMG316372 | HM032748 | *Craniella* sp. 3878 | LCO - cox1 calc R2 |  |
| QMG318785 | HM032752 | *Craniella* sp. 3318 | cox1 calc D1 - cox1 calc R1 | LCO - cox1 R2 Tetillid |
| QMG320143 | HM032746 | *Cinachyrella schutzei* | cox1 calc D1 - cox1 calc R1 | LCO - cox1 calc R1 |
| QMG320270 | HM032741 | *Cinachyrella* sp. 3473 | LCO - cox1 calc R1 | LCO - cox1 tet R11 |
| QMG320636 | HM032745 | *Cinachyrella schulzei* | cox1 calc D1 - cox1 R1 | LCO - cox1 R1 |
| QMG321405 | HM032743 | *Cinachyrella australiensis* | LCO - cox1 calc R1 | LCO - cox1 R2 Tetillid |
| TAU-M0293 | HM032738 | *Cinachyrella alloclada* | LCO - cox1 R1 |  |
| TAU-M0728 | HM032740 | *Cinachyrella* sp. | LCO - cox1 R1 |  |
| TAU-M0761 | HM032739 | *Cinachyrella* sp. | LCO - cox1 R1 |  |
